# Damage Detection Using Ultrasonic Techniques in Concrete-Filled Steel Tubes (CFSTs) Columns

**DOI:** 10.3390/s22124400

**Published:** 2022-06-10

**Authors:** Antonio Callejas, Roberto Palma, David Hernández-Figueirido, Guillermo Rus

**Affiliations:** 1 Department of Structural Mechanics, University of Granada, 18071 Granada, Spain; rpalgue@ugr.es (R.P.); grus@ugr.es (G.R.); 2 Instituto de Investigación Biosanitaria, ibs.GRANADA, 18012 Granada, Spain; 3 Excellence Research Unit “ModelingNature” (MNat), 18071 Granada, Spain; 4 Department of Mechanical Engineering and Construction, Universitat Jaume I, 12071 Castellón de la Plana, Spain; hernandd@uji.es

**Keywords:** nondestructive evaluation, ultrasound, Broadband Ultrasound Attenuation, concrete-filled steel tubes

## Abstract

Concrete-filled steel tubes (CFSTs) are structural elements that, as a consequence of an incorrect elaboration, can exhibit internal defects that cannot be visualized, being usually air voids. In this work, the detection of internal damage in CFST samples elaborated with a percentage of contained air voids in concrete, was carried out by performing a complete ultrasound scan using an immersion tank. The analysis of the ultrasound signals shows the differences presented in the amplitude of the fundamental frequency of the signal, and in the Broadband Ultrasound Attenuation (BUA), in comparison with a sample without defects. The main contribution of this study is the application of the BUA technique in CFST samples for the location of air voids. The results present a linear relationship between BUA averages over the window of the CFSTs and the percentage of air voids contained (Pearson’s correlation coefficient r = 0.9873), the higher percentage of air voids, the higher values of BUA. The BUA algorithm could be applied effectively to distinguish areas with defects inside the CFSTs. Similar to the BUA results, the analysis in the frequency domain using the FFT and the STFT was sensitive in the detection of internal damage (Pearson’s correlation coefficient r = −0.9799, and r = −0.9672, respectively). The results establish an improvement in the evaluation of CFST elements for the detection of internal defects.

## 1. Introduction

The use of Concrete-Filled Steel Tubular (CFST) columns is a popular solution for high rise buildings, in modern bridges, sports stadia, towers, and offshore structures [1]. A CFST column is formed by infilling concrete into a hollow steel tube. The steel tube confines the concrete core and provides a permanent formwork, and the concrete core avoids or delays the local buckling of the steel tube. This combination enhances the structural behaviour of both materials increasing the strength capacity, ductility, and fire resistance of the column which leads to reduced sections and more usable floor area and thus the economic benefit of the building [2]. In addition, CFSTs are fast to erect, and it is possible to work in different levels at the same time due to concrete can be infilled pumping from the bottom. In addition, B.K. Oh et al. [3] demonstrated that CFSTs are more sustainable, in terms of $CO_{2}$ emissions than other traditional solutions. Many studies were carried out on CFST columns subjected to concentric load, eccentric load, impact load [4], stiffened with V-shaped grooves [5], with internal stiffeners [6], or with outer annular stiffener [7], and the behavior of this type of structural elements are well-known. In contrast, a reduced number of studies were carried out to measure the importance of the imperfections in CFST columns.

Two materials are used in CFST columns, and both are prone to imperfections. Imperfections in steel tubes due to the manufacturing process affect the local and global buckling of the columns. In CFST columns, the influence of the concrete core reduces these effects [2]. In concrete, the main problems are related to the influence of concrete compaction during execution, corrosion of steel, working loads, and changes in temperature. Two recent works studied the detection of cracks due to steel corrosion in reinforced cement mortar, one by means of a correlation of the nonlinear elastic features of ultrasonic waves with the critical events of the corrosion [8], and another through intermodulation generation of ultrasonic waves [9]. Some of the typical defects in CFSTs are internal voids and separations between steel and concrete that can be regional or complete. Main defects in the form of spherical gaps between concrete core and steel tube make a negative effect on the confinement contribution causing a decrease of compressive strength of CFSTs by almost 2–14% [10,11,12,13] compared with a healthy specimen, creating a partially confined effect between concrete and steel, that the compressive strength of the CFST is similar to the one of concrete without steel. Air voids located through the matrix of concrete in CFSTs minimize the mechanical contribution of concrete and affect it similarly as the percentage of porosity does, making a decrease of compressive strength when the percentage of porosity increases [12]. Experiments in CFSTs with simulated air voids [14] show an increase in buckling deformation compared to a healthy specimen, and a reduction in its ultimate compressive load and ductility. For this reason, the prevention of failures through the detection of defects is of great importance in the elaboration of CFST elements, and existing structures using CFSTs. A new ultrasonic index were proposed by Chao G. et al. [15,16] to determine the influence of air-void on the final strength of the arch bridge rib of CFST; however, the work did not study the presence of air voids inside the concrete.

CFSTs have the inconvenient that internal defects in concrete cannot be visualized. An approach to detect internal defects is the analysis of ultrasound signals in the time-frequency domain, using several methods such as Fast Fourier Transform [17,18,19], Short-Time Fourier Transform [20,21] or Wavelet Transform [22,23]. Analyzing the spectra of frequencies of ultrasound signals helps in the detection of internal flaws and composition of materials since it is difficult to make a good comparison and a characterization of the ultrasound signals in the time domain in highly attenuated materials, such as concrete. The evaluation of ultrasound signals in the frequency domain to detect damage in concrete is mainly applied since there is a more sensitive change in amplitudes of the signals, attenuation, and energy of harmonics than in the ultrasonic pulse velocity method (UPV), when internal damage (small inclusions, internal voids and cracks) is presented in concrete [22,24]. Evaluation in the frequency domain using the Fast Fourier Transform of ultrasound signals in concrete with internal cracks made by induced damage shows in [19] that a decrease in the energy of the fundamental harmonics appears to be compared to the analysis of ultrasound signals in locations of the same specimen without damage. In addition, in [18] showed that the energy spectra of the fundamental frequency of signals in damaged concrete induced by compression tests decreased significantly compared to the healthy elements, although UPV remained with almost the same values. In experimentations evaluating concrete with induced porosity by freeze-thaw cycling and salt-scaling in [17], UPV decreased just 3–9% compared to a healthy specimen, and values of the amplitude of the fundamental harmonic, evaluating the spectrum of the Fourier Transform, resulted in a more sensitive parameter than a change of UPV, presenting higher values of energy of the fundamental frequency of ultrasound signals through healthy concrete specimens than in damaged ones.

Analysis of ultrasound signals through the Wavelet Transform decomposes the signal into a specific wave-like oscillation (wavelet), different from the Fourier Transform that makes a decomposition of the signal into sine waves of different frequencies. Short-Time Fourier Transform and Wavelet Transform are usually applied in pulse-echo technique, since the detection of echoes caused by internal flaws, when ultrasound signals are submerged in noise, can be done evaluating the signals in the time-frequency domain [25]. Even though, an investigation of interest [23] of a concrete structure with holes located in different depths, demonstrated that the UPV, using the through-transmission technique, does not have a linear relation of the size of the internal hole, since the time of flight of the ultrasonic pulse of the signals traveling through an internal hole were similar to the ones in the intact concrete, but there was a clear difference in the location in time and amplitude of the energy of the fundamental frequency evaluating the received signal in the time-frequency domain using a combination of the Short-Time Fourier Transform and the Wavelet Transform, called S Transform. Similar results were obtained in a simulation study carried out by Nadom K. et al. [26]. The detection of damage and its size is subject to the change in the colour scheme of the STFT spectrogram. The resultant images show that the increase in the frequency of the excitation signal gives better results.

Broadband Ultrasound Attenuation (BUA) is a physically meaningful way of obtaining attenuation of material as the slope of the linear regression over a certain range of frequencies. BUA was introduced initially by Langton et al [27], applied in medical experimentations to determine the effects of attenuation, scattering, and porosity of cancellous bones. The BUA is obtained by measuring the difference of the spectra of an ultrasonic wave transmitted through reference material, such as water, and through the material to be analyzed [28]. The difference of both spectra results in an attenuation versus frequency curve, being the BUA the slope of this curve in a certain range of frequency. As R. Strelitzki et al. [29] mentioned, a certain range of frequencies could be selected for the BUA regression slope, usually a range between 200 kHz to 1MHz, depending on the fundamental resonant frequency of the transducer used, being in most of the case the selected range of frequencies the one that best fits a linear regression. Even though it is possible that different materials have similar BUA values, this parameter can be used to do a comparison between healthy and unhealthy samples.

BUA parameter is commonly used in experimentations in the field of bioengineering applied to identify properties of bones [30] as differences between healthy bones and cancellous bones [31,32,33], and relationships between BUA parameter and porosity of bones [34], but there are some references of experimentations done in other materials. J.B Hull et al. [35] made an important approach of BUA applied in the identification of polymer materials and porosity of ceramics. In this experimentation, they performed a relation of the attenuation versus frequency regression slope (BUA) with the time of flight of the ultrasound signal through the material to obtain Hull/Langton index (HL), used to make a comparison with porosity in ceramics measured with hydration test, resulting in a linear relationship of HL index increasing as the percentage of porosity in ceramics does. BUA is shown in [36] that can be applied in the same way for detection of defects (as drill holes) in aluminum bars making an interesting approach on how BUA has higher values when a material is analyzed in a fully drilled area than in an area with smaller drill holes, applied with both pulse-echo and through-transmission methods. A comparative result [37] in mortars with different degrees of porosity demonstrated that mortars with a higher percentage of porosity have higher regression slope values (BUA) in the attenuation versus frequency curve for a certain range of frequencies (between 1.5 to 1.7 MHz) than mortars with less percentage of porosity. Although the degree of porosity cannot be obtained from BUA results, there is an approach about how BUA values allow differentiating between an element that has a percentage of damage.

Based on the studies presented, there is a knowledge gap about the effectiveness of the different analysis methods that can be applied to the existing ultrasound techniques for the detection of damage, specifically air voids inside the concrete matrix of CFSTs. The objective of this study is to measure possible internal defects in the concrete. In this line, CFST stub columns with internal defects were manufactured including random inclusions.

The remainder of this paper is organized as follows. Section 2 describes the preparation of samples, the experimental setup, and the algorithms employed to detect internal damage in CFSTs. Section 3 describes the results and the discussion. Finally, Section 4 offers the conclusions of this research and suggestions for the future.

## 2. Materials and Methods

### 2.1. Preparation of Samples

**Figure 1:**
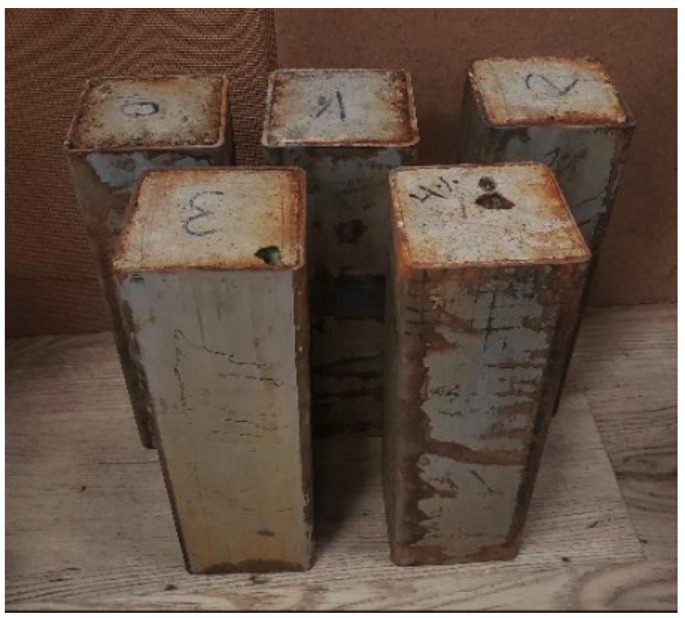
CFST stub columns.

In this work, a total of 5 CFST stub columns were tested to detect internal imperfections. All these columns were manufactured in the laboratory of the Department of Mechanical Engineering and Construction of the Universitat Jaume I in Castellón, Spain. The columns were 300 mm in length with a nominal cross-section of the tubes (height (h) × width (b) × steel thickness (t)) 100 × 100 × 4 mm (see Figure 1). The steel tubes were cold-formed carbon steel and supplied by the same manufacturer. The nominal yield strength of the tubes was S275JR.

Air voids were simulated using a certain amount of expanded polystyrene beads with a volume of 25 mm in diameter. This material is conformed to 98% of air, for this reason, expanded polystyrene pieces in the concrete can be simulated as internal air voids. The percentage of voids for each CFST is 0%, 1%, 2%, 3% and 4%. The approximate number of air voids can be estimated with the previous information, as seen in Table 1.

Elaboration of concrete, fully described in [38] (proportions, Table 2), was done using a planetary mixer to prepare the concrete mix. Concrete and polystyrene pieces were poured in steel tubes by stages using a vibrator rod to compact concrete correctly. A concrete specimen was used to obtain the values of the characteristic resistance. Concrete and CFSTs were standardly cured for 28 days covered with wet clothes, then external surfaces were treated to have a proportional dimension.

### 2.2. Experimental Setup

**Figure 2:**
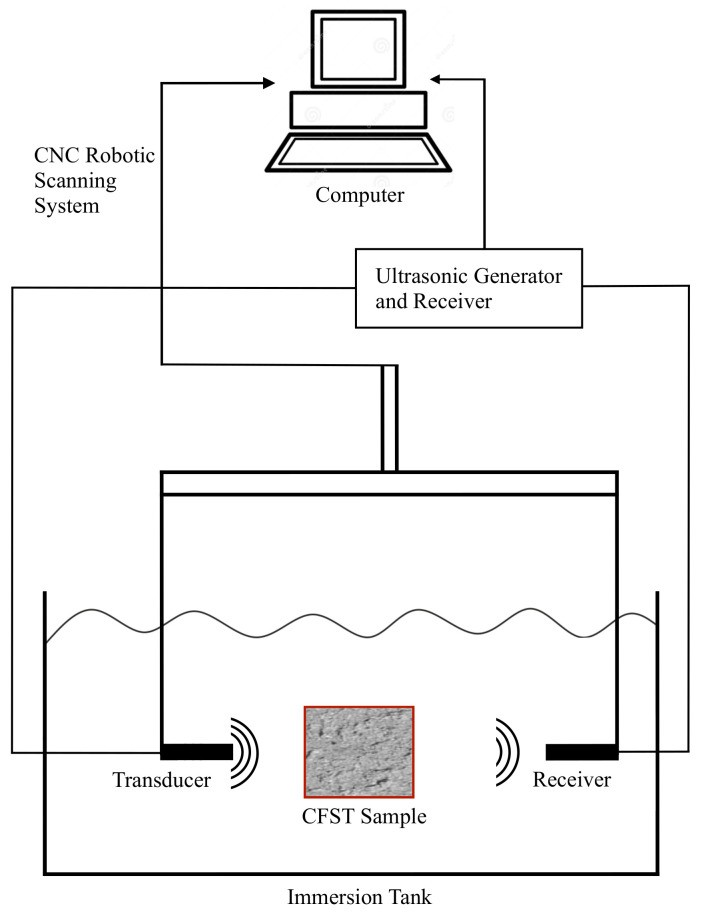
Experimental setup.

**Figure 3:**
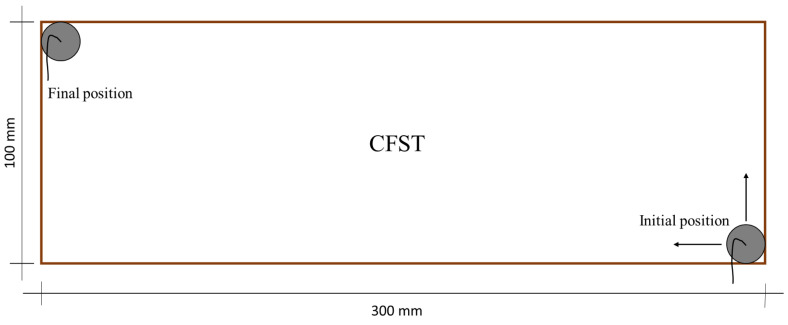
Schematic path of transducers during the scanning process. The gray circles represent the diameter of the transducers. The arrows show the directions of movement of the same.

The through-transmission technique was used to perform a 2D scan of the specimens in an immersion tank. Figure 2 shows a controlled arm used to correct alignment between the transducers and the specimens and to scan them without changing the distance emitter-receiver. Taking into account the compromise of the attenuation of waves with the frequency, immersion transducers with a central frequency of 1 MHz (0.5”-V303) were selected. The transducer with a smaller diameter was used to increase the resolution in the 2D scan. The maximum voltage registered by the receiver was obtained by rotating the transducers on their vertical and horizontal axis. The distance from the emitter transducer to the CFST samples was set to avoid near-field effects. In this case, the separation was 30 mm. The receiver was positioned at 50 mm from the CFST sample to prevent reflections from interfering with the recorded signal. The edge of the transducer was aligned to the lateral edge of the CFST samples, as shown in Figure 3. The ultrasonic device was programmed to perform measurements every 2 mm in the two dimensions of the movement.

An ultrasonic testing device with integrated pulser and receiver was employed (OPBOX 2.1). A maximum voltage of 360 V was set and the gain pre-amplifier of +24 dB was used. An analog filter of 0.5–25 MHz was applied. Signals were acquired with a sampling frequency of 100 MHz, and an averaging of 64 samples was set.

### 2.3. Computational Algorithms

#### 2.3.1. Fast Fourier Transform and Short-Time Fourier Transform

Transformation of the signals in the frequency domain was done using the Fast Fourier Transform (FFT) algorithm in Matlab (R2018b, The MathWorks Inc., Natick, MA, USA). To prevent leakage and aliasing, 8192 signal points were employed.

Short-time Fourier transform (STFT) is a sequence of Fourier transforms of a windowed signal. STFT provides the time-localized frequency information for situations in which frequency components of a signal vary over time. STFT algorithm of an ultrasound wave signal s(t) can be written as,

(1) $$S\left(\tau ,\omega \right)=\int_{-\infty }^{+\infty }\;s\left(t\right)\theta \left(t-\tau \right)e^{-i\omega t}\;dt$$

where θ(t−τ) is the window function with time and duration, τ is the time resolution, and ω is the radial frequency. Here, a 512-point Hamming window with 50% overlap was employed. The spectrogram allows to represent the signal in the time-frequency domain and evaluate the evolution of the frequencies of the signal over time to detect, in a more precise way, differences between ultrasound signals as its phase velocity and the energy spectrum of the fundamental frequency emitted by the transducer.

In STFT analysis, there exists a tradeoff between time and frequency resolution when determining the window size. In other words, although a narrow-width window results in a better resolution in the time domain, it generates a poor resolution in the frequency domain, and vice versa.

#### 2.3.2. Broadband Ultrasound Attenuation Analysis

**Figure 4:**
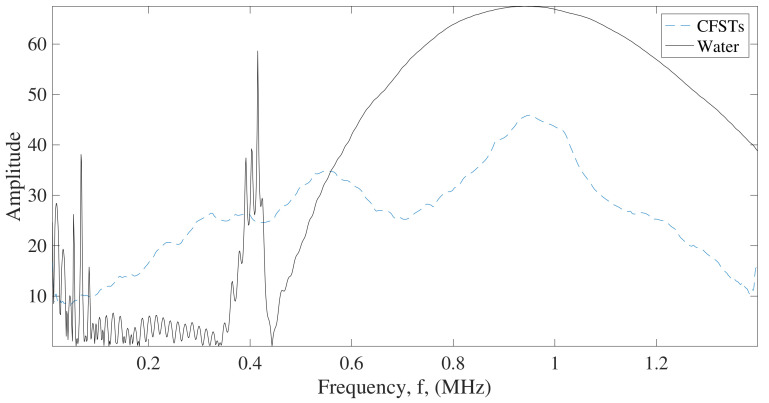
Representation of frequency spectra of reference material (degassed water) and CFST sample (dashed line).

The attenuation parameter to compare intact and damaged structures was obtained by comparing the frequency spectrum of the transmitted signal through a reference material that does not contain defects or has low attenuation and the spectrum of the analyzed specimen to be characterized (Figure 4). In this work, degassed water was used for reference signal.

Attenuation at a certain frequency is the difference between the amplitudes of both spectra, the reference material, and the characterized material [29]. Therefore, to obtain the attenuation parameter, the subtraction of the absolute values of the spectra of the reference material (VR), obtained from the FFT, and the absolute values of the spectra of the material to be characterized (VS) should be obtained as,

(2) $$\alpha =20\cdot Log_{10}\left|VR\right|-20\cdot Log_{10}\left|VS\right|$$

where α is the attenuation.

**Figure 5:**
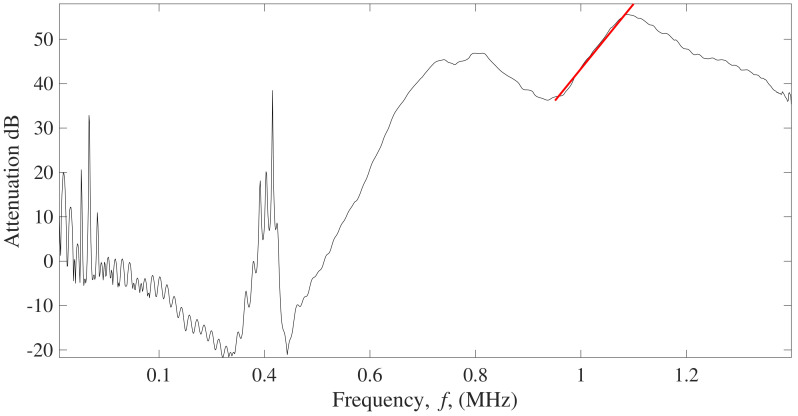
Example of the attenuation versus frequency curve (black solid line) with regression slope (red solid line).

The BUA parameter was determined by fitting the attenuation values within a selected frequency range to a straight line (Figure 5) as follows,

(3) α=a+BUA·f

where *f* is the frequency in MHz and *a* is the intersection value of the curve with the vertical axis. The range of frequencies to obtain BUA regression slope highly depends on the fundamental resonant frequency of the transducers used, in this case, the transducers used were 1 MHz. The range of frequencies to obtain BUA rate was from 0.95 MHz to 1.1 MHz (see Figure 5) because it is the range of frequencies around the fundamental frequency of the transducer and that the variation of the attenuation as a function of frequency has a linear behavior.

## 3. Results and Discussion

### 3.1. FFT and STFT C-Scan Results

**Figure 6:**
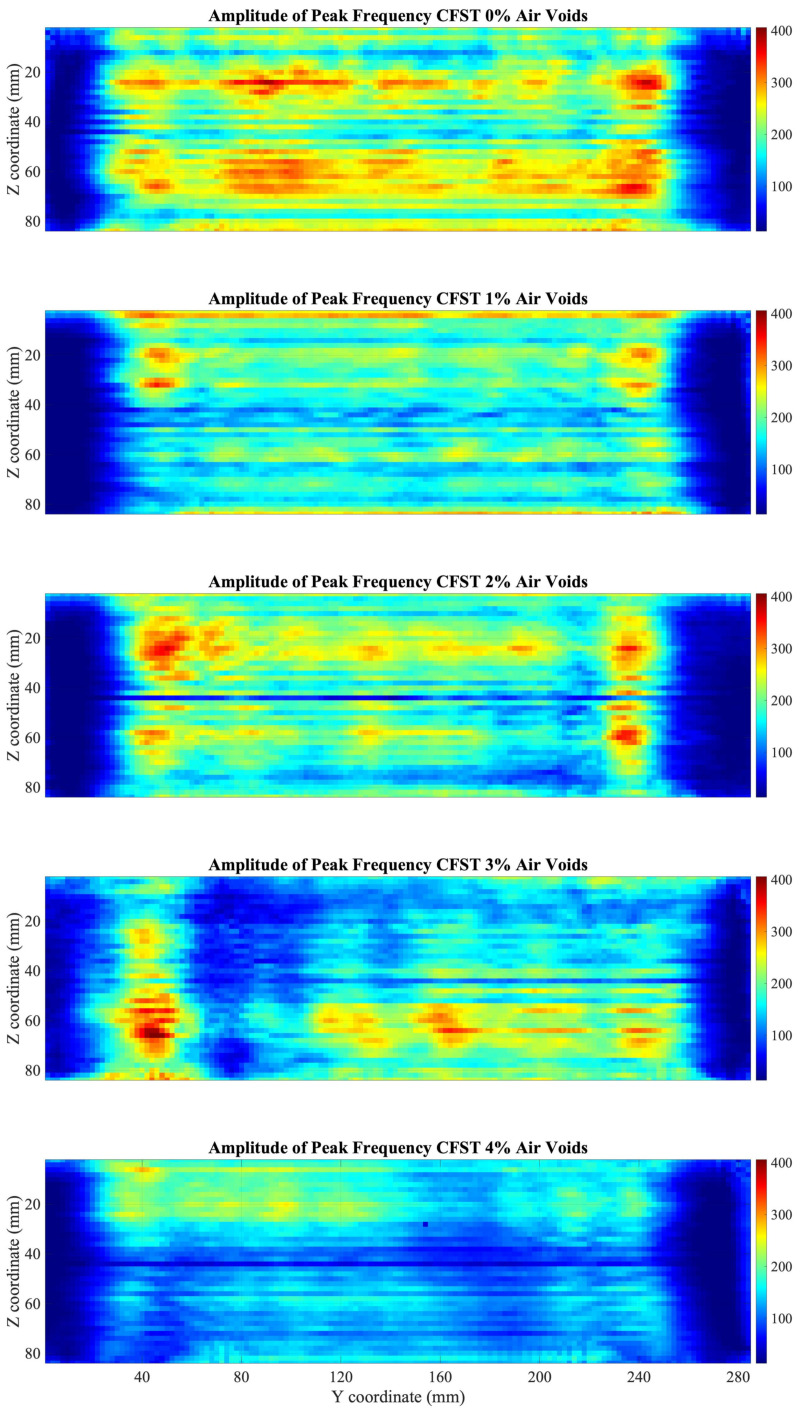
C-Scan of fundamental harmonic amplitudes using FFT for the five CFST samples.

The FFT algorithm was applied to the received signals of the 5 CFST samples, and a C-Scan was obtained evaluating the amplitude of the fundamental harmonic of every signal. Results in Figure 6 present a clear difference of the energy spectrum of the signals in the CFST samples according to the percentage of air voids contained.

As shown in Figure 6, a decrease in the amplitude of the fundamental frequency is exhibited on the sides of the CFST samples, compared with values at the center of the CFST. This drop in energy is given due to the high attenuation of the signals in this area since part of the energy from the transducer travels through the water. As the transducer moves away from the edges this effect disappears.

**Figure 7:**
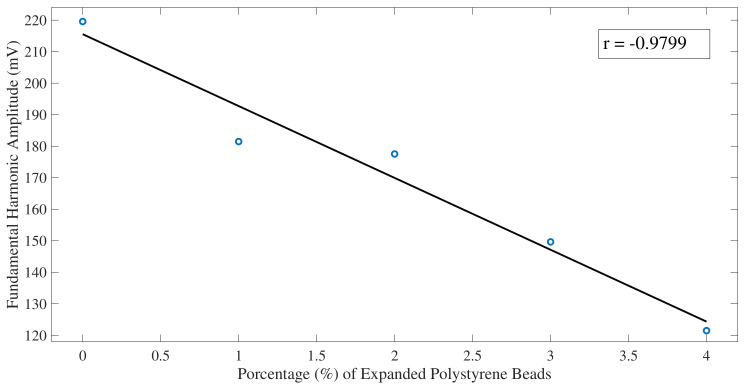
Fundamental harmonic amplitude averages over the window of the CFST samples using the FFT algorithm. The solid black line is a linear fit of the data. Pearson correlation coefficient r = −0.9799.

Average of the fundamental harmonic amplitudes, excluding the lateral part of the CFST sample (30 mm on each side) (see Figure 7) shows a decrease of the amplitude of the fundamental harmonic energy as the percentage of air voids contained in the CFSTs increments.

From the obtained result of the fundamental harmonic amplitude averages over the window of the CFST samples, it is shown that the average energy value decreases as the percentage of polystyrene beads increases.

Similarly, the algorithm of STFT was applied to the signals of the 5 CFST samples using the parameters described previously. The C-Scan was performed evaluating the highest value of the energy spectrum, close to 1 MHz.

**Figure 8:**
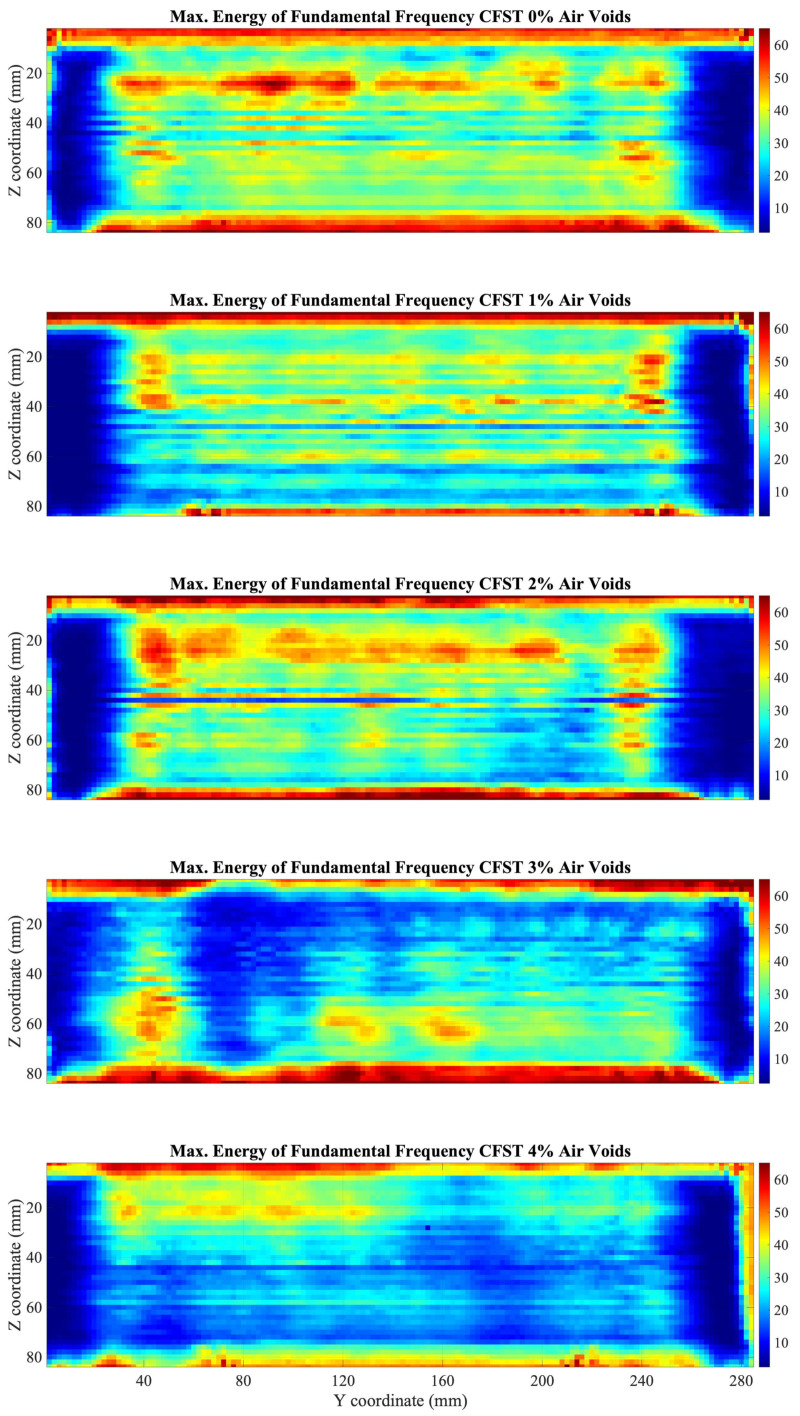
C-Scan of the fundamental harmonic amplitudes using STFT for the five CFST samples.

Although similar results are presented in Figure 8, in comparison with the use of the FFT algorithm, they present some differences. As seen in the C-Scan, in the top and bottom of the CFST, there is an increment in the amplitude of the fundamental frequency due to the signals transmitted through steel. In addition, the range of the energy spectrum is considerably less than the obtained using the FFT algorithm.

**Figure 9:**
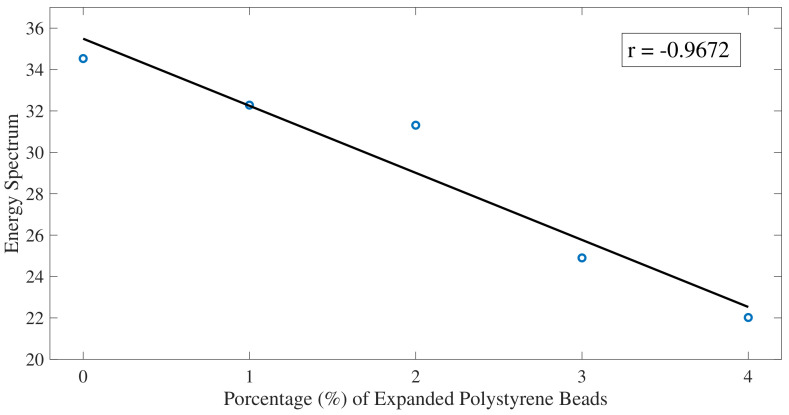
Fundamental harmonic amplitude averages over the window of the CFST samples using the STFT algorithm. The solid black line is a linear fit of the data. Pearson correlation coefficient r = −0.9672.

The results of the STFT analysis show, as with the FFT algorithm, a reduction in the average value over the analyzed window as the percentage of gaps increases (Figure 9), motivated by the fact that the ultrasounds when traveling through the polystyrene beads reduce the amplitude considerably due to the difference in impedance between two media.

Assumptions of the areas of damaged concrete, due to air voids contained, could be done. The air voids that are contained in the lateral parts of the CFST cannot be visualized, since at these positions the wave travels through the CFTS sample and part through the water.

The analysis in the frequency domain using the FFT and the STFT was sensitive in the detection of internal damage. When transforming the signals in the frequency domain using the FFT a decrease in the amplitude of the fundamental frequency was presented if the signal travels through an air void. Similar results were displayed evaluating the time-frequency energy spectrum using the STFT where a reduction of the level of energy of the fundamental frequency was presented. C-Scan using previous algorithms allowed to identify damaged areas in the CFST samples. Results were validated since the average values of both C-Scan presented a decrease in the energy of the fundamental frequency as the percentage of air voids increased. Several works presented in the literature to detect internal defects in the time-frequency domain showed similar results, such as Fast Fourier Transform [17,18,19], and Short-Time Fourier Transform [20,21]. Analyzing the frequency spectrum helps in the detection of internal flaws and composition of materials since it is difficult to make a good comparison and a characterization of the ultrasound signals in the time domain in highly attenuated materials, such as concrete.

### 3.2. BUA C-Scan Results

**Figure 10:**
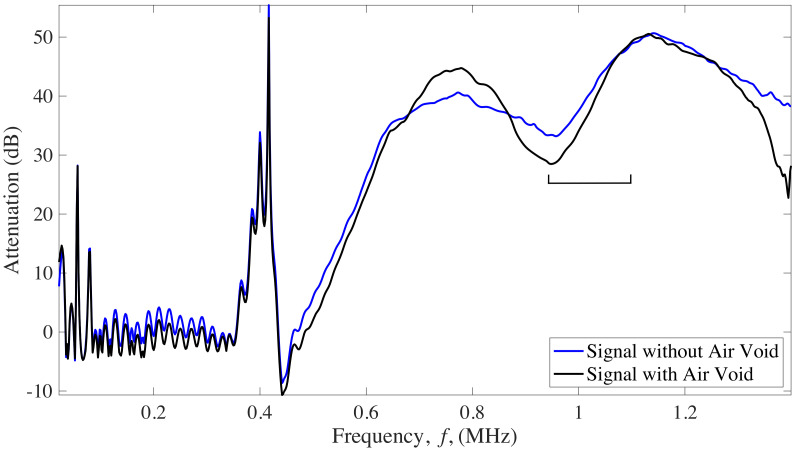
Attenuation versus frequency curve from ultrasound signals measured in the 5 CFST samples. The square bracket indicates the frequency range in which the linear regression is performed.

First, from the obtained signals in the five different CFST samples, a comparison between the attenuation versus frequency curves obtained from signals taken at the center of the CFST sample was done. As shown in Figure 10, the linear regression can be applied in the same range of frequencies (950 kHz to 1.1 MHz) to obtain a difference in the BUA values that allow making a distinction between signals that pass through an air void, and from signals passing through an intact area of the CFST.

From the previous result, it can be distinguished that, in the attenuation versus frequency curves a higher slope (BUA) is presented in signals that travel through air voids than in those that travel through the concrete. Therefore, a C-Scan of the BUA values was performed to detect the damaged areas.

**Figure 11:**
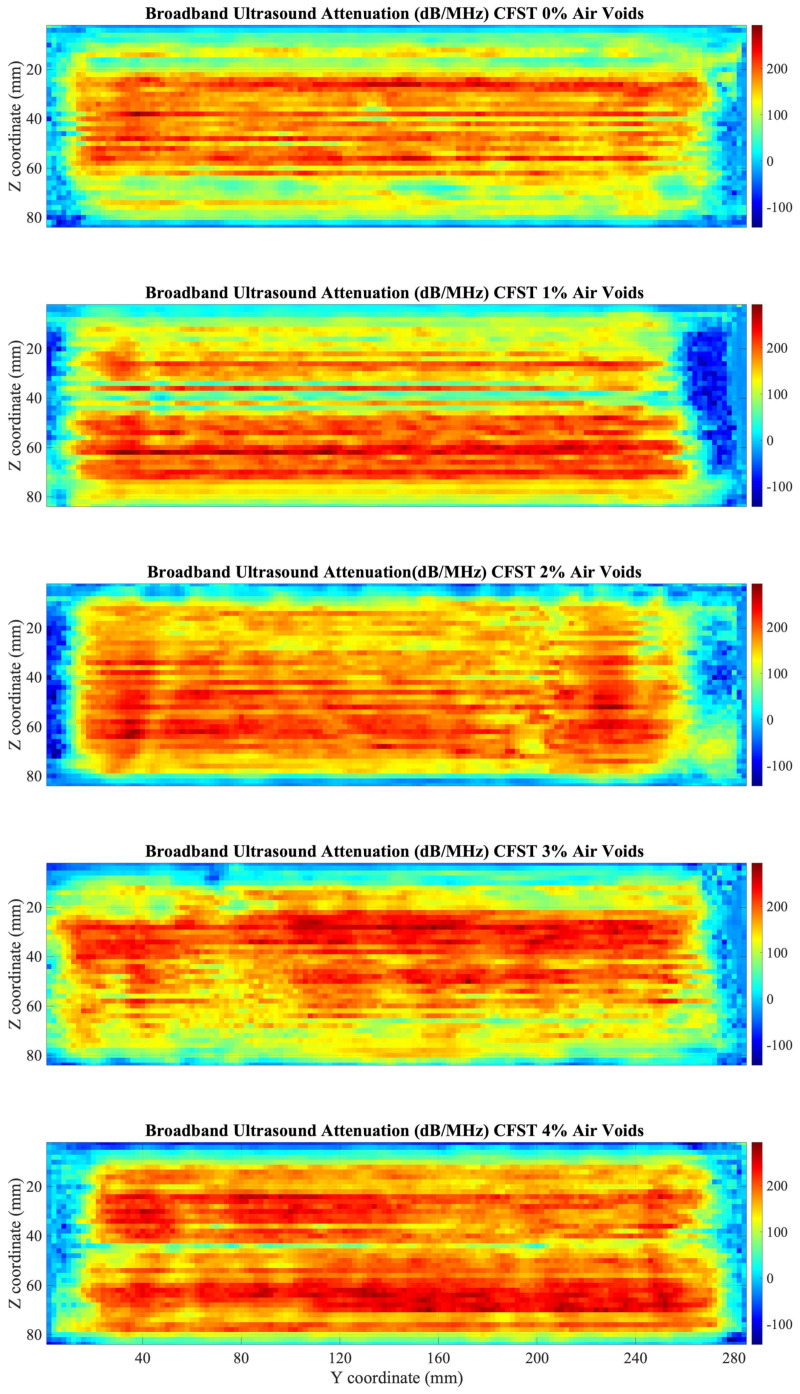
C-Scan of BUA values in dB/MHz for the five CFST samples.

**Figure 12:**
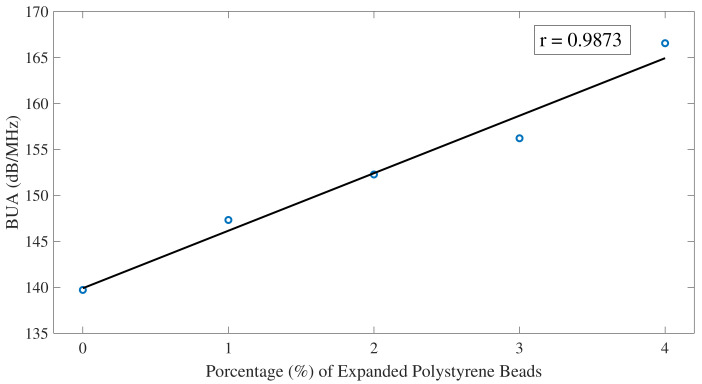
BUA averages over the window of the CFST samples. The solid black line is a linear fit of the data. Pearson correlation coefficient r = 0.9873.

Results from the BUA C-Scan are presented in Figure 11. Quantification of results was obtained with averages of the BUA parameter over the window of the CFST samples, and results were plotted against the percentage of air voids, as shown in Figure 12. Results, although affected by the lateral parts of the CFSTs, present a linear relationship between BUA and the percentage of air voids contained, the higher percentage of air voids, the higher values of BUA.

**Figure 13:**
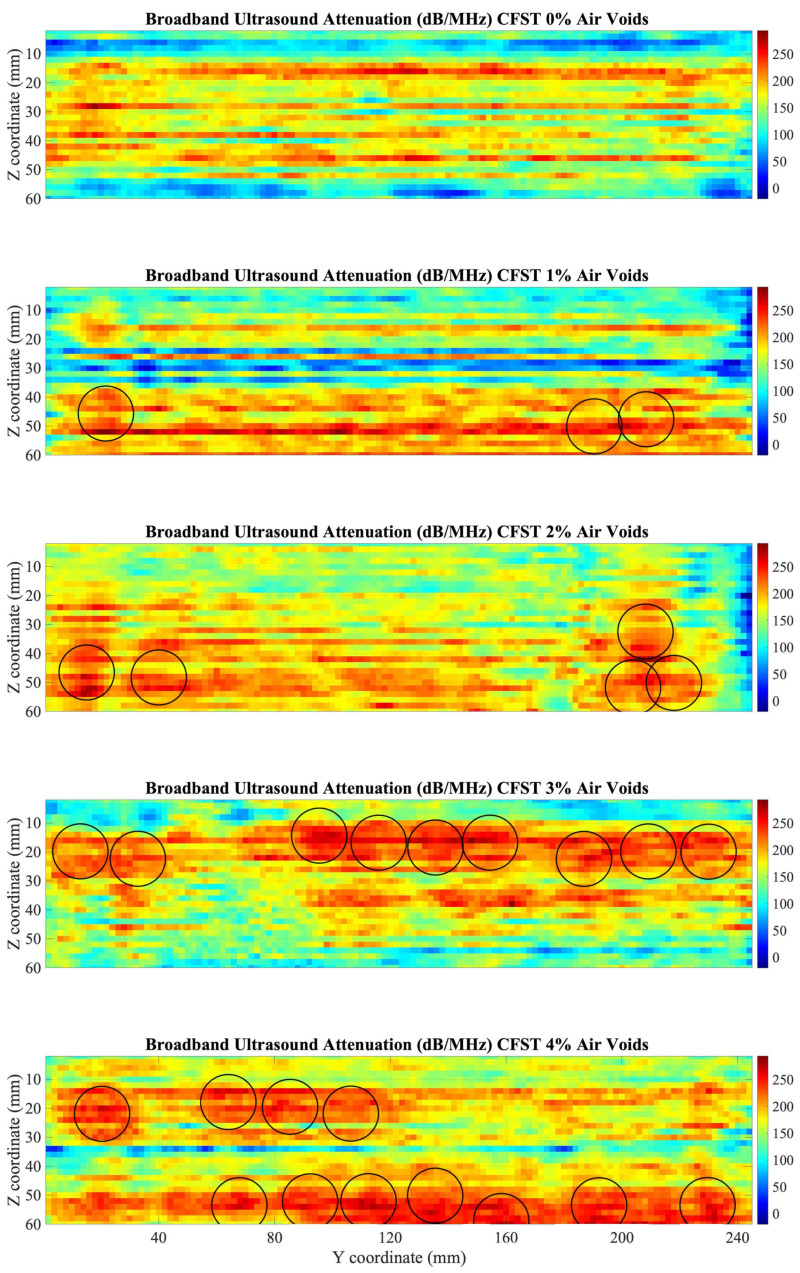
C-Scan of BUA values in dB/MHz at the center of the five CFST samples. The circles represent the position of the air voids calculated by the proposed algorithm.

Similar to frequency domain analysis results from the lateral parts of the CFST are different from the values at the center of the CFST samples. For a better comparison of the C-Scan, results excluding the lateral and upperparts that affect the range of BUA values were analyzed (15 mm on each side, and 10 mm top and bottom were excluded), as seen in Figure 13.

Damage can be detected in a more accurate way area in the CFSTs samples (Figure 13), allowing to do an estimation of the location of the expanded polystyrene beads included in the elaboration of the CFST samples. For that purpose, a damage detection algorithm was programmed in Matlab environment. The algorithm performs a sweep over the entire C-scan. In each of the positions, a circle of the diameter of the polystyrene beads is positioned. A BUA threshold is established and if the average of the values inside the circle is greater than the determined threshold, that zone is identified with an inclusion. The set threshold was 240 dB/MHz, obtained through iteration to capture the estimated approximate number of voids in each CFST sample (see Table 1). Figure 13 shows the reconstruction of the air voids, which, in general, are aligned with the longitudinal direction of the samples, which makes sense due to the introduction of a vibrator rod for sample compaction.

Results of analyzing the signal through BUA indicate that this method could be used to detect defects inside CFSTs. The selected range of frequencies to analyze BUA was from 950 kHz to 1.1 MHz, which has a relation with the central frequency of the transducers used. As mentioned by R. Strelitzki et al. [29] there is certain range of frequencies that could be selected for the BUA regression slope, depending on the fundamental resonant frequency of the transducer used, being in most of the case the selected range of frequencies the one that best fits a linear regression. This frequency range was analyzed since more differences were presented in the regression slope of the attenuation versus frequency curve if an ultrasound signal travels through an area with defects. BUA averages over the window of the CFST samples presented a linear increase with the percentage of air voids contained in the sample. Similar results were found in mortars with different degrees of porosity [37], mortars with a higher percentage of porosity have higher regression slope values (BUA) in the attenuation versus frequency curve for a certain range of frequencies than mortars with less percentage of porosity. Although the degree of porosity cannot be obtained from BUA results, there is an approach about how BUA values allow differentiating between elements with different percentage of damage. In addition, the work performed by Rosalba et al. [36] showed that BUA has higher values when aluminum bars are analyzed in a fully drilled area than in an area with smaller drill holes, applied with through-transmission methods.

Results from Pearson’s correlation study were shown in Figure 7, Figure 9, and Figure 12. Pearson’s correlation coefficient r = −0.9799 for the FFT study, r = −0.9672 for the STFT study, and r = 0.9873 for the BUA study. Despite that the three studies show high correlation coefficients, the BUA study is the one that shows the highest value (r = 0.9873). Despite this higher correlation, the edge effects of the C-scans (left and right areas) in both the FFT and STFT methods (Figure 6 and Figure 8) mask information related to possible concrete imperfections; however, with the BUA method (Figure 11) these edge effects are reduced, obtaining more information in the mentioned areas.

## 4. Conclusions

Concrete-filled steel tubes (CFSTs), formed by infilling concrete into a hollow steel tube, are structural elements that can present internal defects such as air voids. In this work, the nondestructive evaluation using the analysis of ultrasound signals in the frequency domain was presented. The Fast Fourier Transform, Short-Time Fourier Transform, and Broadband Ultrasound Frequency techniques were employed. The detection of internal damage was carried out by doing a complete ultrasound C-Scan in the CFST samples using an immersion tank. Five CFST stub columns were tested, which have a certain percentage of failure being this 0%, 1%, 2%, 3% and 4%, based on the percentage of expanded polystyrene beads added to the concrete specimen, simulating air voids, in comparison with the volume of concrete. For the three proposed techniques, fundamental harmonic amplitude (FFT and STFT techniques) or BUA averages over the window of the CFST samples were performed. The results present a linear relationship between BUA averages over the window of the CFSTs and the percentage of air voids contained (Pearson’s correlation coefficient r = 0.9873), the higher percentage of air voids, the higher values of BUA. The BUA algorithm could be applied effectively to distinguish areas with defects inside the CFSTs. Similar to the BUA results, the analysis in the frequency domain using the FFT and the STFT was sensitive in the detection of internal damage (Pearson’s correlation coefficient r = −0.9799, and r = −0.9672, respectively). When transforming the signals in the frequency domain using the FFT a decrease in the amplitude of the fundamental frequency, corresponding to the central frequency of the transducer (1 MHz), was presented if the signal travels through an air void. Similar results were displayed evaluating the time-frequency energy spectrum using the STFT where a reduction of the level of energy of the fundamental frequency was presented. In summary, the results of this investigation establish an improvement in the evaluation of CFST elements for the detection of internal defects. As future research works, based on the limitations of this work, a study of the samples is proposed using the pulse-echo technique, to solve the inspection difficulties by requiring two accessible faces; as well as 2D scanning with smaller diameter transducers to achieve higher resolution and to be able to inspect defects at the edges between the concrete and the steel.

## Figures and Tables

**Table 1:** Approximate number of added polystyrene beads.

Percentage of Air Voids	Polystyrene Beads
0%	0
1%	3
2%	6
3%	9
4%	12

**Table 2:** Concrete mix proportions.

Infill	Proportions
Cement (kg/m${}^{3}$)	348
Water (l/m${}^{3}$)	220
Sand (kg/m${}^{3}$)	1065
Gravel (kg/m${}^{3}$)	666

## Abbreviations

The following abbreviations are used in this manuscript:

CFSTs	Concrete-filled steel tubes
BUA	Broadband Ultrasound Attenuation
CFST	Concrete-Filled Steel Tubular
UPV	Ultrasonic pulse velocity method
HL	Hull/Langton index
FFT	Fast Fourier Transform
STFT	Short-time Fourier transform
